# Optimization of a fatty acid methyl ester protocol for quantification of odd- and even-chain fatty acids in yeast

**DOI:** 10.1186/s13568-026-02022-8

**Published:** 2026-02-09

**Authors:** Veronica Bonzanini, Otto Savolainen, Sergio Morales Palomo, Majid Haddad Momeni, Lisbeth Olsson, Cecilia Geijer

**Affiliations:** 1 Division of Industrial Biotechnology, Department of Life Sciences, Chalmers University of Technology, Chalmersplatsen 4, 412 96 Gothenburg, Sweden; 2 AAK AB, Pulpetgatan 20, 215 37 Malmö, Sweden; 3 Chalmers Mass Spectrometry Infrastructure (CMSI), Chalmers University of Technology, Chalmersplatsen 4, 412 96 Gothenburg, Sweden

**Keywords:** Triacylglycerol (TAG), Gas chromatography-mass spectrometry (GC–MS), Oleaginous yeast, Odd-chain fatty acid (OCFA), Even-chain fatty acid (ECFA), Deuterated internal standard, Microbial oils

## Abstract

Fatty Acid Methyl Ester (FAME) analysis is widely applied in fields ranging from food and biofuel research to fatty acid profiling in microorganisms. Given the challenges associated with accurately quantifying both even- and odd-chain fatty acids, this study presents a tailored FAME protocol employing a fully deuterated internal standard (dIS) for their simultaneous analysis. Two oleaginous yeast species, *Yarrowia lipolytica* and *Blastobotrys adeninivorans*, were chosen for their distinct fatty acid profiles and served as models to evaluate protocol sensitivity and extraction efficiency. Here, we compared two simultaneous extraction and transesterification methods, a microwave-assisted and a shaker-assisted protocol, and found that the microwave-assisted approach not only successfully recovered the dIS but also extracted yeast fatty acids more efficiently than the shaker-assisted method. Incorporating a bead-beating pre-extraction step further improved lipid recovery and reduced variability. Our results suggest that the non-methylated dIS, when added as a free fatty acid, methylates more slowly than yeast-derived fatty acids that are mainly found as triacylglycerols. This can complicate normalization and supports the use of a pre-methylated dIS. While FAME yields varied depending on the extraction method and applied normalization strategies, the FAME profiles remained consistent across protocols, highlighting strong reproducibility in qualitative analyses. The optimized FAME protocol provides a precise and reliable analysis of fatty acids in diverse oleaginous yeast species. Our results underscore the need for species-specific optimization of FAME protocols and warn against relying on a deuterated single fatty acid standard without protocol adjustment.

**Supplementary Information:**

The online version contains supplementary material available at 10.1186/s13568-026-02022-8.

## Introduction

Microbial production of renewable oils is gaining global attention as a sustainable alternative to traditional plant, animal and fossil-based oil resources, with particular focus on oleaginous yeasts that, by definition, can accumulate more than 20% of their total dry weight in lipids. Most yeast lipid research focuses on even-chain fatty acids (ECFAs), whereas odd-chain fatty acids (OCFAs) are generally overlooked by standard assays and considered to occur in only trace amounts. However, OCFAs like pentadecanoic acid (C15:0), heptadecanoic acid (C17:0) and heptadecenoic acid (C17:1) are increasingly recognized for their valuable medical and nutritional applications. There is evidence that insufficient dietary intake of OCFAs is linked to an increased risk of developing conditions such as obesity, chronic inflammation, cardiovascular diseases, type 2 diabetes, and certain cancers (Forouhi et al. 2014; Khaw et al. 2012; Kurotani et al. 2017; Venn-Watson et al. 2020; Venn-Watson and Schork 2023). Therefore, there is a drive to expand our understanding and identify and analyze the entire fatty acid profiles including the OCFAs across different oleaginous yeast species.

**Fig. 1:**
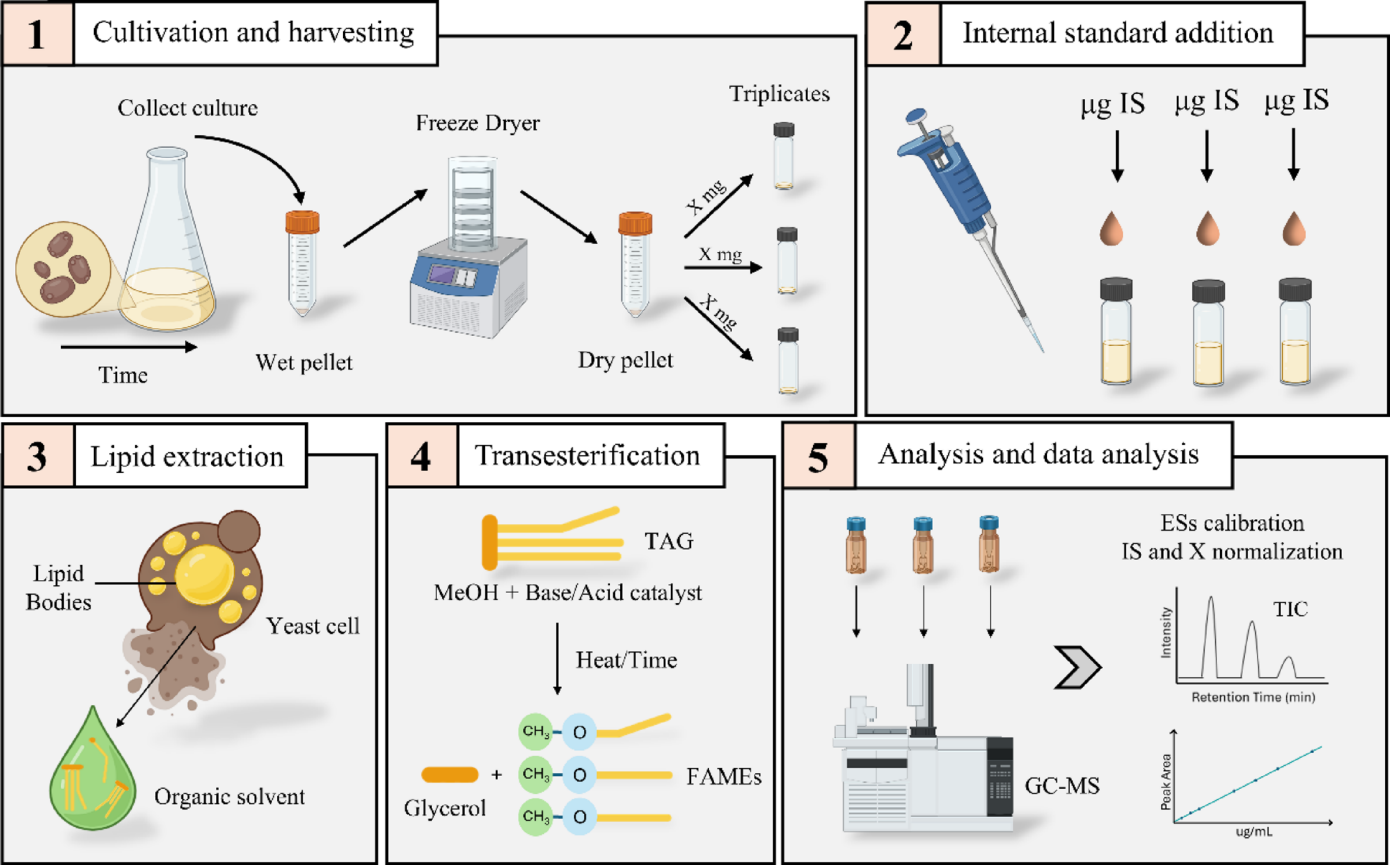
Procedure for a typical FAME experiment. Five steps are illustrated by: **1** Cultivation and harvesting of samples, including freeze drying of the biomass to remove residual water that can influence the FAME protocol, and weighing a defined amount of biomass in triplicates for subsequent lipid extraction. **2** Addition of a defined amount of the selected internal standard, to track losses and for normalization. **3** The lipid extraction process, which can be performed with different techniques and in presence of an organic solvent to dissolve the TAGs and extract them from the complex matrix. **4** Transesterification of the extracted triacylglycerols which represent the core reaction of the protocol to convert the fatty acids into fatty acid methyl esters, generating also glycerol. **5** The final analysis using GC–MS, followed by external standard calibration for quantification, data processing and normalization. The figure was generated with BioRender.com ES, external standards; IS, internal standard; TAG, triacylglycerol; TIC, total ion chromatogram.

Fatty acid analysis is widely applied across diverse fields, including food quality control, nutritional assessment, human health studies, biofuel evaluation, and the profiling of various organisms. Given the substantial variation in sample matrices, it is crucial to develop and optimize protocols tailored to specific applications to ensure proper lipid extraction and purification before analysis. To quantify and profile fatty acids produced by yeasts, researchers typically use a Fatty Acid Methyl Eester (FAME) protocol (see Fig. 1 for an overview of the method). First, oleaginous yeasts are cultivated under experimental conditions of interest, after which the cell biomass is harvested and freeze-dried (Fig. 1, step 1). The composition of the produced fatty acids is strongly influenced by the yeast species as well as cultivation parameters, including process design and medium composition (Bonzanini et al. 2025).

The second step in the procedure is the addition of a suitable internal standard (IS) that is compatible with the protocol (Fig. 1, step 2). Internal standards play a crucial role in fatty acid analysis by GC–MS, as they account for sample preparation and pipetting errors, injection inconsistencies, and instrumental variations. In microbial oil research focusing on ECFAs, OCFAs such as heptadecanoic acid (C17:0) or nonadecanoic acid (C19:0) are often used as internal standards (Vasconcelos et al. 2018; Williams et al. 2021). For OCFAs analysis, internal standards in the form of OCFAs are unsuitable since the internal standard must differ from the target analytes. Instead, a deuterated internal standard (dIS) can be used to ensure accurate quantification. DISs are stable isotope-labeled compounds in which hydrogen atoms are replaced with deuterium (^2^H or D), retaining close to identical chemical and ionization properties to their non-labeled counterparts (Wang et al. 2017). In mass spectrometry, however, they can be distinguished by their *m/z* value and often elute at slightly different retention times in the total ion chromatogram, usually earlier than the non-deuterated analogue (Sparkman et al. 2011). DISs are typically used as single fatty acids, which get converted (esterified) into FAME alongside the transesterification of the yeast-derived fatty acids that are mainly in the form of TAGs, thereby accounting for experimental losses and, to some extent, transesterification efficiency. In contrast, if the dIS is added as a FAME (i.e. pre-methylated), it only compensates for experimental losses, not for reaction efficiency. Regardless of the approach, protocols incorporating deuterated compounds as ISs must be carefully optimized and validated to ensure reliable quantification and effective normalization.

The third critical step in FAME analysis is the extraction of fatty acids from yeast cells, mainly in the form of TAGs (Fig. 1, step 3). Yeasts represent a highly diverse group of organisms, with considerable variation in morphology and cell wall structure, which can lead to markedly different responses to extraction treatments (Jacob 1992; Zainuddin et al. 2021). Moreover, the extracted sample matrices are often complex, containing different types of lipids, proteins and oligosaccharides, potentially affecting the extraction and therefore precision and accuracy of the FAME protocol. Intracellular TAGs from yeasts can be extracted using various methods, including mechanical techniques (such as bead beating, sonication, or microwave-assisted extraction) and non-mechanical methods, which can be physical, chemical, or biological (such as osmotic shock, acid/base and enzymes) (Zainuddin et al. 2021). It is important to note that none of the methods described allow for standardized TAG extraction from yeast using intracellular controls, which negatively impacts accuracy.

In the fourth step, extracted TAGs should be transesterified into FAMEs, where several experimental options are available (Fig. 1, step 4). The most common approach involves a reaction between the TAGs and methanol to produce FAMEs (Knothe et al. 2005). Transesterification also requires a catalyst to accelerate the reaction, with typical catalysts being alkaline, acids, or enzymes (Nisar et al. 2021). Additionally, solvents such as hexane are often added to facilitate the extraction of FAMEs from the sample matrix. Key parameters influencing the efficiency of the transesterification process include the molar ratio of alcohol to TAGs, reaction temperature, time, and moisture content (Knothe et al. 2005). In several protocols described in literature, lipid extraction and transesterification are carried out simultaneously (Khoomrung et al. 2012; Qiao et al. 2015).

The fifth and final step is the separation, identification, and quantification of the produced FAMEs, typically performed using gas chromatography–mass spectrometry (GC–MS) technique (Fig. 1, step 5). Here, effective peak separation is essential to avoid co-elution, which can compromise both qualitative and quantitative accuracy, particularly when analyzing structurally similar fatty acids. Moreover, to ensure robust quantification and to correct for variability in sample handling, derivatization, and GC–MS analysis, the internal standard is used for normalization. This approach enables comparison of fatty acid levels across different samples, experiments and experimental conditions.

Accurate analysis of ECFAs and OCFAs, including those present only in trace amounts, requires careful understanding, evaluation, and optimization of all five steps of the FAME protocol. This study focuses on evaluating and optimizing key steps such as lipid extraction, FAME transesterification methods, including the selection and application of a dIS, and GC–MS analysis, with the aim of achieving satisfactory peak separation. Given that using different yeast species may influence the analytical outcomes, two phylogenetically distinct oleaginous yeasts were compared in this study: *Yarrowia lipolytica*, known for its high lipid accumulation capacity (Ledesma-Amaro and Nicaud 2016), and *Blastobotrys adeninivorans* (formerly *Arxula adeninivorans*), which typically produces lower amounts of lipids (Bonzanini et al. 2025). 

## Materials and methods

### Yeast strains, media and cultivation

The yeast strains used in this study were *Yarrowia lipolytica* W29 (Westerdijk Fungal Biodiversity Institute, Utrecht, Netherlands (CBS)) and *Blastobotrys adeninivorans* Y-17692 (National Center for Agricultural Utilization Research, Illinois, USA (NRRL)). The strains were preserved in glycerol stocks with a final concentration of 25% v/v glycerol in sterile 96-well plates and stored at −80 °C.

The pre-culture medium contained a low molar carbon to nitrogen ratio (C/N ratio 9) and consisted of 1.7 g/L YNB (Yeast Nitrogen Base) without amino acids and ammonium sulfate (filter sterilized), 20 g/L glucose, and 5 g/L (NH_4_)_2_SO_4_. Also 50 mM phosphate buffer prepared from 28.2 g/L Na_2_HPO_4_ and 11.45 g/L KH_2_PO_4_ was used to keep pH at 6.8. The yeasts were inoculated directly from glycerol stocks into sterile culture tubes with 15 mL of pre-culture medium and grown until reaching stationary phase (about 48 h) at 30 °C, 200 rpm.

The strains were cultivated as single replicates in 250 mL flasks with 85 mL of medium similar to the pre-culture medium, but with a molar C/N ratio of 50, achieved by maintaining 20 g/L of glucose and reducing the (NH_4_)_2_SO_4_ to 0.89 g/L. The flasks were inoculated with a final OD_600_ = 0.1 and incubated at 200 rpm and 30 °C. The biomass samples were taken at the end of cultivation (115 h), washed in MilliQ water once, decanted, frozen at −80 °C overnight and freeze dried. To ensure that differences observed in downstream analyses reflected methodological performance rather than biological variation, a single homogeneous biomass batch was generated per strain. The biomass was freeze-dried, aliquoted and analyzed in technical triplicates for each method. This experimental design allowed a direct comparison of analytical methods using identical biological material from two yeast strains, but did not capture biological variability between independent cultivations of the same strain.

### Internal and external standards

The internal and external standards used in this study are the same as those employed in our previous work (Bonzanini et al. 2025). The selected internal standard is a fully deuterated myristic acid (d27-C14:0). The free fatty acid form was obtained from Merck (Cat. No. 366889, Darmstadt, Germany), and the pre-methylated form was purchased from Nordic Biosite (Cat. No. 154-28594-50, Täby, Sweden). For quantitative analysis, the pre-methylated external standards include a range of OCFAs and ECFAs, spanning from C6:0 to C24:1, encompassing both saturated and unsaturated forms. The OCFA methyl ester standards were sourced from Larodan (Solna, Sweden), and the ECFA methyl ester standards were obtained from Nu-Check Prep Inc. (Minnesota, USA). Concentrated stock solutions of all internal and external standards were prepared in hexane and stored at −20 °C in solvent-safe tubes. For a comprehensive list of external standards, see Table 1 in reference (Bonzanini et al. 2025). For calibration purposes, pre-methylated external and internal standards were run alongside the samples in every GC–MS analysis. These standards were prepared as one solution which were then diluted to seven different concentration levels, resulting in final concentrations of 10, 25, 50, 100, 250, 500, and 1000 μg/mL of total fatty acid methyl esters. The calibration range varied depending on the fatty acid analyzed, spanning from 0.4 to 160 μg/mL. Calibration curves were generated using the GC-Solution software by correlating total ion chromatogram peak areas with the corresponding concentrations. Following calibration, the amount of internal standard detected in the sample was used for normalization, as described in equation [1] below.

**Table 1:** Comprehensive information of key steps of microwave- and shaker-assisted protocols

Protocol steps	Microwave-assisted	Shaker-assisted
Starting biomass	Freeze dried (20 mg)	Freeze dried (20 mg)
Extraction	Simultaneous to TE	Simultaneous to TE
Solvents	Hexane:Methanol 1:2	Hexane:Methanol 1:1
Catalyst	BF_3_	NaOH
Trans-esterification	MW1 or MW2	Vortex 1200 rpm, 1 h at RT
Addition	Milli-Q water	49% v/v H_2_SO_4_
Phases separation	Centrifuge	Centrifuge

RT, room temperature; TE, trans-esterification; MW, microwave

### Lipid extraction and transesterification

Two different protocols for lipid extraction and transesterification were compared (see Table 1 for an overview):

A “shaker-assisted protocol” was adapted from a previous study (Qiao et al. 2015). Briefly, 20 mg of freeze-dried biomass samples were resuspended in 500 µL of methanol (MeOH) solution containing 1 M sodium hydroxide (NaOH), which represents the reagent and base catalyst for transesterification. The MeOH-NaOH solution was prepared by dissolving NaOH in MeOH overnight with continuous stirring. Moreover, 98 µg of myristic acid (d27-C14:0) dIS dissolved in hexane was also added to the samples. The sample mixtures were vortexed at 1200 rpm at room temperature for 1 h. Afterward, 80 µL of sulfuric acid (49% v/v) and 500 µL of hexane were added to the mixture to neutralize the excess base and convert any free fatty acids into their methyl esters, followed by centrifugation at 2000 rpm for 2 min. The upper hexane layer was carefully recovered and diluted as necessary before analysis by GC–MS.

**Fig. 2:**
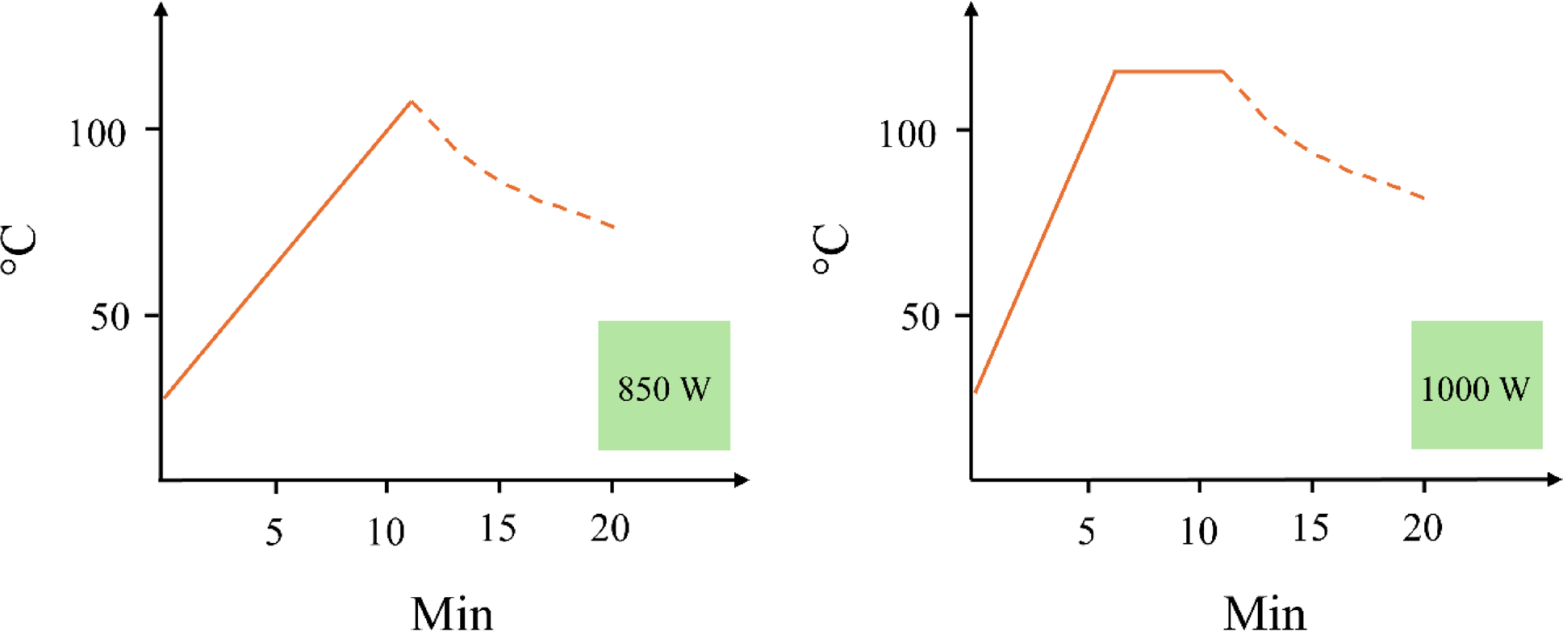
Schematic representation of the two microwave (MW) methods tested in this study. Panel **A** shows MW1, operated at 850 W, which represents the slower method. The temperature gradually increased from room temperature to 110 °C over 11 min. Panel **B** shows MW2, operated at 1000 W, which is the more intense method. Here, the temperature is ramped from room temperature to 120 °C in 6 min, followed by a 5-min hold at 120 °C. The dotted line illustrates the cooling phase of the system

The “microwave-assisted protocol” was adapted from another study (Khoomrung et al. 2012) with minor modifications. Briefly, samples of 20 mg freeze-dried biomass were combined with 1 mL of hexane and 2 mL of 14% w/v BF₃ in MeOH with the addition of 98 µg of myristic acid (d27-C14:0) dIS dissolved in hexane. The air in the tubes were purged out with nitrogen (N₂) flushing for 30 s, followed by vortexing for 20 s. The TAG extraction and transesterification steps were performed simultaneously using a microwave digestion system (Milestone Start D, Sorisole Bergamo, Italy). Two different microwave (MW) methods, processing 19–22 vessels per run (full microwave loading), were tested. In method 1 (MW1), the microwave was set to 850 W and the heating gradient increased from room temperature to 110 °C over 11 min, while in method 2 (MW2), the microwave was set to 1000 W and the temperature ramped gradually from room temperature to 120 °C within 6 min and was held for 5 min (Fig. 2). In both methods, sealed capsules containing 30 mL of water housed the sample tubes inside.

After microwave processing, 2 mL of Milli-Q water was added and mixed with each sample, followed by phase separation via centrifugation at 2500 rpm for 5 min. The entire upper hexane phases containing the extracted FAMEs were carefully transferred to GC vials, dried under a nitrogen (N₂) flow, and resuspended in 1 mL of hexane, whereafter the samples were diluted as necessary and analyzed by GC–MS. In the final test of the optimized protocol, the step of hexane evaporation and subsequent resuspension of the extracted FAMEs was omitted, and the sample, following dilution, were loaded directly into GC–MS after microwave treatment.

To test lipid extraction efficiency, a mechanical pre-extraction step for disrupting cells using glass beads was introduced prior to the microwave-assisted protocol to increase lipid extraction recovery. The extraction process was performed as described in a previous study (Bonzanini et al. 2025). Briefly, 300 µL of glass beads (425–600 μm, acid washed with 0.1 M HCl), 500 μL of hexane, and 98 μg of deuterated myristic acid (d27-C14:0) as internal standard were added to the freeze-dried biomass. Cell disruption and TAG extraction were performed using Fast Prep (six cycles at 8000 rpm for 30 s and 2 min pause intervals) followed by centrifugation (2000 rpm for 5 min). The hexane phase was then recovered and transferred to new Pyrex glass tubes for subsequent steps in the microwave-assisted protocol. This pre-extraction step has been performed in all the subsequent experiments.

### GC/MS analysis for fatty acid determination

The analysis was carried out in a GC–MS single quadrupole (QP2020 NX from Shimadzu, Japan), using standards as described above and in (Bonzanini et al. 2025). Column, injection mode, carrier gas, and the mass transfer line are consistent with those described previously (Bonzanini et al. 2025). The initial oven method (standard operating procedure at Chalmers Mass Spectrometry Infrastructure) runs for 12 min: 80 °C with a 2-min hold, followed by a ramp to 160 °C at 40 °C/min, then to 185 °C at 5 °C/min, and finally to 260 °C at 30 °C/min, with a final 0.5-min hold. The optimized oven method runs for 25.58 min: 50 °C with a 2-min hold, followed by a ramp to 160 °C at 10 °C/min, then to 185 °C at 3 °C/min, and finally to 260 °C at 20 °C/min, with a final hold of 0.5 min. Means and standard deviations were calculated from three technical replicates.

FAME yield in µg/mg = mg/g of biomass with dIS normalization was calculated as follows, using calibration with external standards provided in µg/mL:

1 $$FAME \left(\frac{\mu g}{mg X}\right)= \frac{\left(\frac{FAME \left(\frac{\mu g}{mL}\right)}{dIS \left(\frac{\mu g}{mL}\right)} \bullet {dIS}_{added} (\mu g)\right)\bullet Df}{X (mg)}$$

where FAME (µg/mL), fatty acid methyl ester concentration after calibration; dIS (µg/mL), deuterated internal standard found in the sample after calibration; dIS_added (µg), initially added dIS amount; Df, dilution factor which corrects for any sample dilution; X (mg), initial biomass amount of sample. Regardless of whether the dIS was added to the sample as non-methylated or as pre-methylated, the calculation remained the same: the amount of dIS determined from calibration in the sample was used to normalize for losses during the protocol, and in the case of the free fatty acid, should also theoretically account for transesterification efficiency.

FAME yield without dIS normalization were calculated as follows:

2 $$FAME \left(\frac{\mu g}{mg X}\right)= \frac{\left(FAME \left(\frac{\mu g}{mL}\right)\bullet V (mL)\right)\bullet Df}{X (mg)}$$

where FAME (µg/mL), fatty acid methyl ester concentration after calibration; V (mL), volume of hexane in which the FAME are dissolved; Df, dilution factor which corrects for any sample dilution; X (mg), initial biomass amount of sample. It should be noted that no conversion from FAME to free fatty acid concentrations, accounting for differences in molecular weight, was applied. Therefore, all results reported in this study are expressed as FAMEs.

### Statistical analysis

F-test two samples were used to find equal or unequal variances, followed by a two-sample student T-test to assess the significance of differences in fatty acid levels between the microwave-assisted protocol with and without the bead-based extraction step, and in the reproducibility test between run 1–2 and run 3–2. Significance levels were set as follows: * < 0.05, ** < 0.01 and *** < 0.001. To assess protocol precision, we analyzed three different batches, each consisting of three technical replicates processed in three independent MW runs. The coefficient of variation (% CV) was calculated for each fatty acid replicate, both within a batch and across batches, as indicated in equation [3]. Finally, the average % CV across all batches was determined.

3 $$CV \left(\%\right)=\left(\frac{Standard Deviation}{Mean}\right) \bullet 100$$

## Results

### Development of GC–MS method for optimal separation of OCFAs and ECFAs

**Fig. 3:**
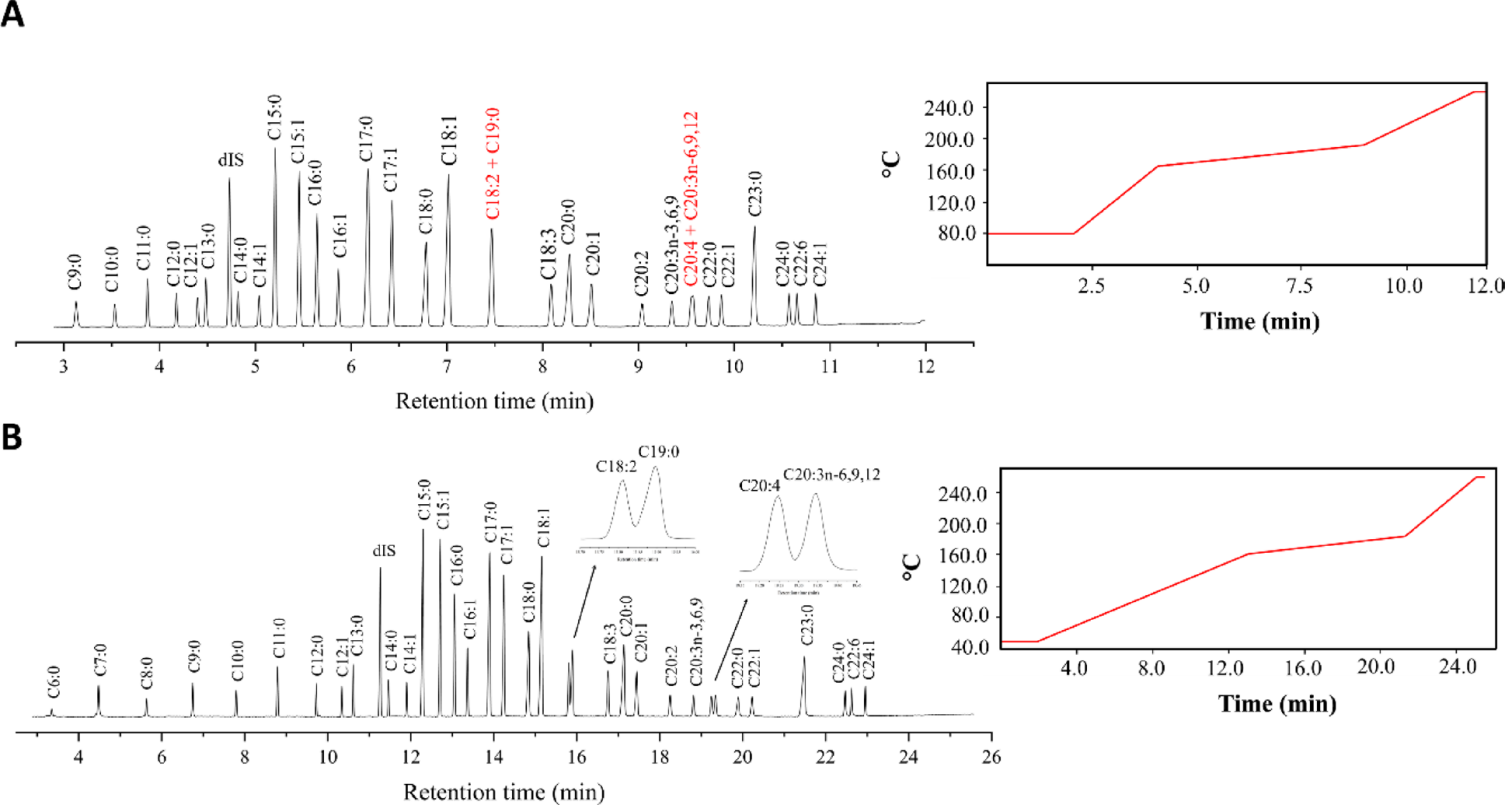
Comparison of GC oven methods and peak separation. Panel **A** schematically illustrates the temperature ramp of the standard GC oven protocol, which has a total runtime of 12 min, along with the corresponding peak separation in the total ion chromatogram. Co-eluting peaks are highlighted in red. Panel **B** shows the temperature ramp of the optimized GC oven protocol, with a total runtime of 25.58 min, and the resulting peak separation in the total ion chromatogram. In both cases the solvent cut time is 2.8 min. The x-axis represents retention time (minutes)

We first set out to establish a reliable GC–MS method that allows for separation of peaks for relevant ECFAs and OCFAs, as it provides the analytical foundation to evaluate the FAME protocol. We prepared a standard solution containing FAMEs derived from both OCFAs and ECFAs, as well as the pre-methylated dIS, and analyzed it using GC–MS following a standard protocol established by the Chalmers Mass Spectrometry Infrastructure for fatty acid analysis (Materials and Methods and Fig. 3A). While most peaks were well resolved under these conditions, the protocol failed to detect short-chain fatty acids (C6:0, C7:0, C8:0) and did not achieve sufficient separation between C18:2 and C19:0, nor between C20:4 and C20:3n-6,9,12.

To address these limitations, the temperature gradient was systematically optimized by adjusting the ramp rates, resulting in a GC–MS protocol that successfully resolved all OCFAs, ECFAs, and the dIS, ranging from very short-chain (C6:0) to very long-chain fatty acids (C24:1). Importantly, the optimized method achieved clear separation of C18:2 from C19:0 and C20:4 from C20:3n-6,9,12 (Fig. 3B). The method provides high-quality analysis of yeast lipids, albeit with a longer run time (close to 26 min instead of 12 min), and it was used consistently in all further analyses.

### Deuterated internal standard is compatible with the microwave-assisted protocol

For initial testing of compatibility of the dIS with lipid extraction and transesterification processing, we cultivated *Y. lipolytica* and *B. adeninivorans* at a C/N ratio of 50 for 115 h to promote lipid accumulation and production of sufficient biomass for downstream analysis. Following biomass harvest, a non-methylated dIS was added to assess its compatibility with two different lipid extraction and fatty acid transesterification protocols: the microwave- and shaker-assisted methods.

**Fig. 4:**
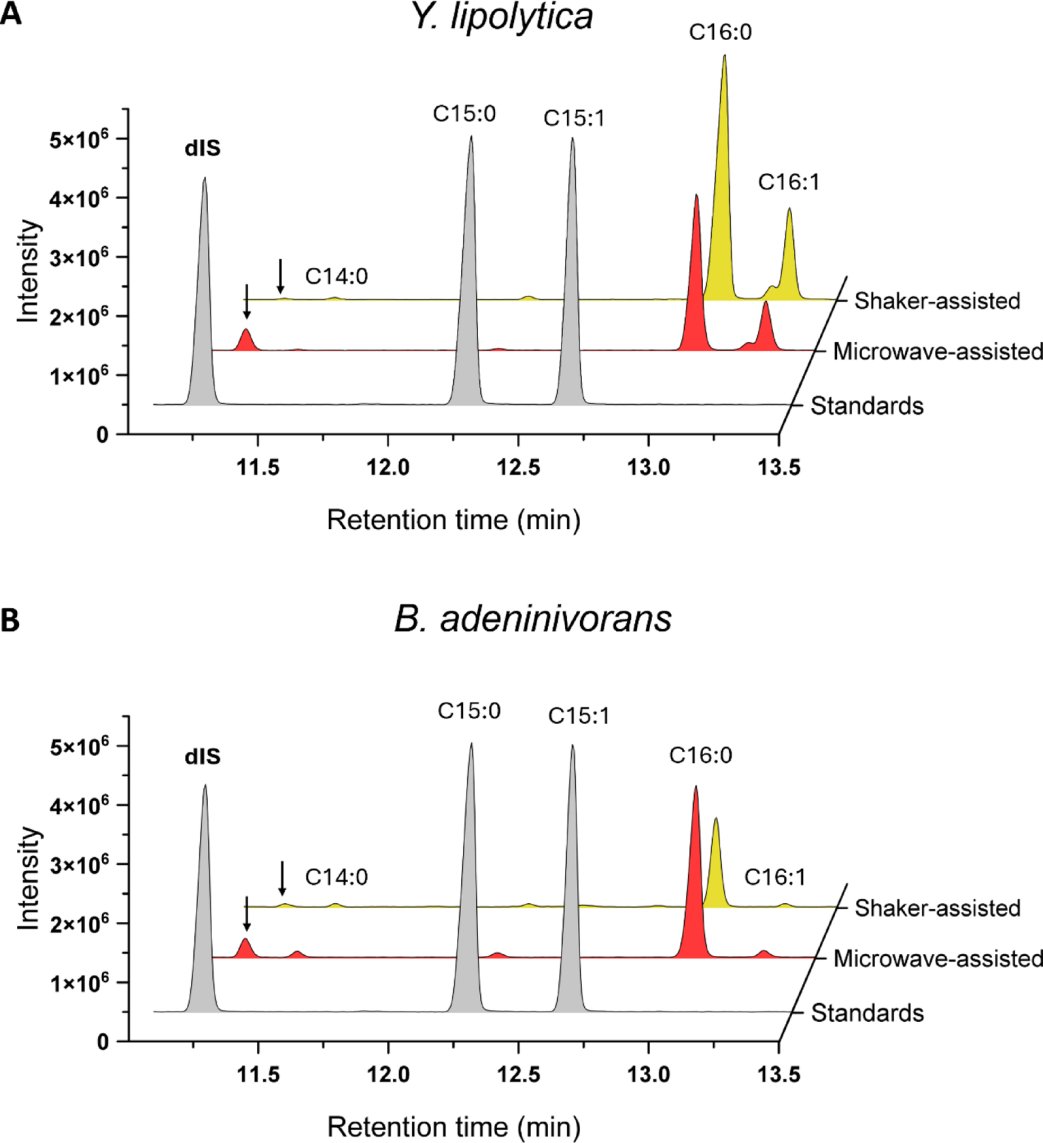
Comparison of a portion of the total ion chromatogram between protocols and strains. Panel **A** illustrates the chromatograms for *Y. lipolytica*, and Panel **B** *B. adeninivorans*. The overlapping chromatograms represent a part of the full chromatograms, depicting the dIS peaks and the two following FAME peaks as reference scale. The chromatogram includes OCFAs external standards and the dIS in gray, sample from the microwave-assisted protocol in red (diluted five-fold for GC–MS run), and sample from the shaker-assisted protocol in yellow (undiluted). One representative sample of the replicates for each protocol is shown in the graph. The x-axis represents retention time in minutes; the y-axis represents the intensity of the peaks (arbitrary unit)

GC–MS analysis of samples from both methods showed that the dIS was clearly methylated and detected using the microwave-assisted protocol, while it was undetectable in the shaker-assisted samples (Fig. 4). Moreover, the microwave-extracted samples required several-fold dilutions for maintaining proper peak shape and reliable detector response in GC–MS analysis, whereas shaker-extracted samples could be analyzed undiluted. This indicates that the shaker protocol was less effective at extracting and/or transesterifying TAGs, and incompatible with dIS esterification. Based on these initial results, we concluded that the microwave-assisted protocol was superior and should be used in subsequent experiments.

### A bead-assisted pre-extraction step improves TAGs extraction

One challenge with the microwave-assisted protocol was that biomass samples frequently seemed to burn during processing in the microwave, likely due to the high temperatures and long exposure time, which resulted in a brown coloration of the solution and compromised sample integrity. To address this, and to see if we could improve the extraction efficiency of the protocol, we introduced a pre-extraction step involving bead beating and mechanical disruption, followed by centrifugation and separation of the lipid-rich hexane phase from the cell debris, prior to microwave treatment.

**Fig. 5:**
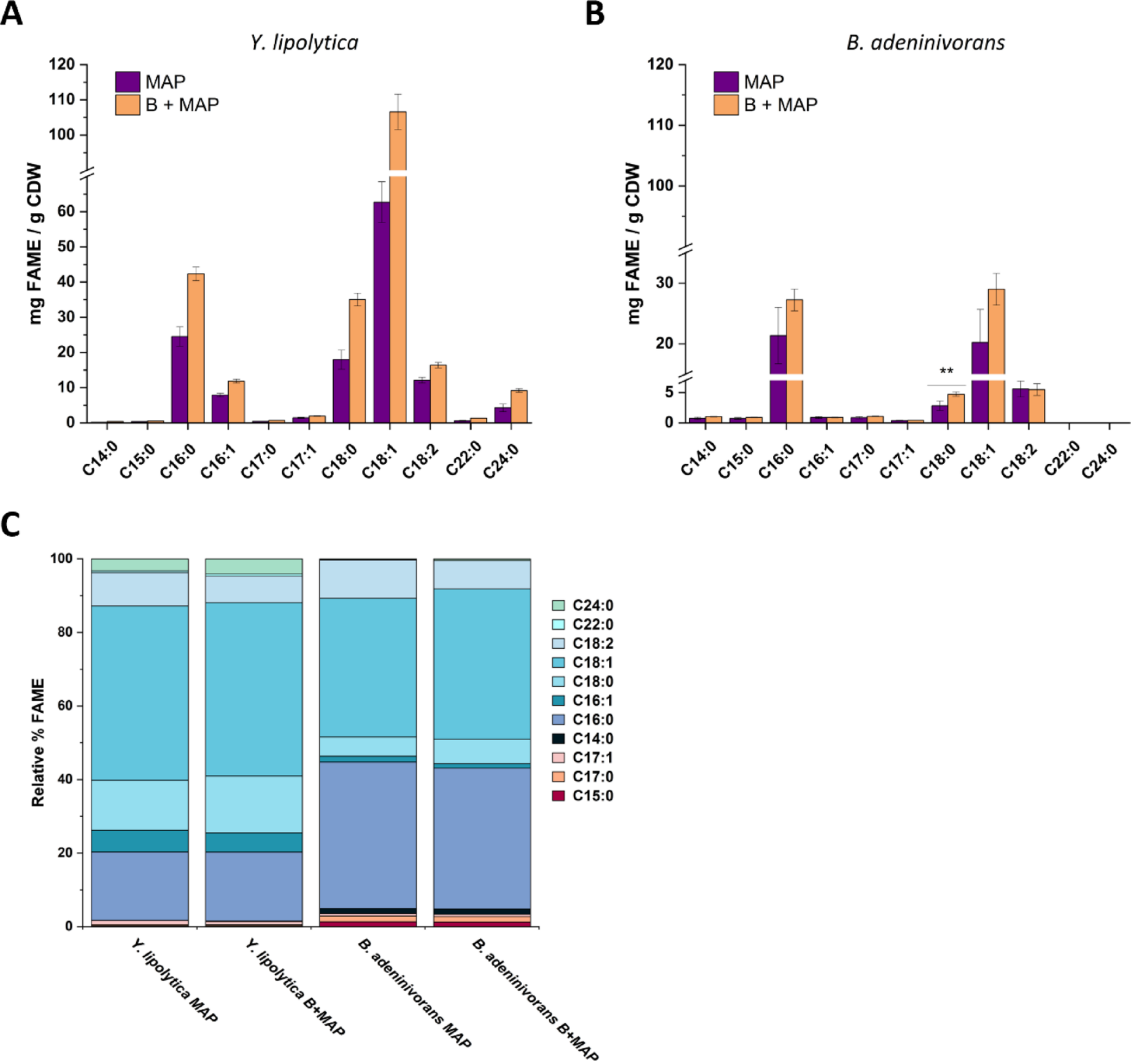
Comparison of extraction efficiencies with and without addition of pre-extraction in the microwave-assisted protocol. Panel **A** displays *Y. lipolytica* and panel **B** *B. adeninivorans*. The x-axis in A and B displays the identified FAMEs in the yeast profiles, while the y-axis shows their yields in mg per g of cell dry weight (CDW). The microwave-assisted protocol (MAP) is shown in purple, and the modified protocol incorporating bead-assisted pre-extraction (B + MAP) is shown in orange. Panel **C** presents the relative percentage composition of different FAMEs for both strains under both conditions (MAP and B + MAP). In panel **A** all the FAMEs in the two conditions are significantly different with a *p* < 0.01. p* < 0.05, ** < 0.01 and *** < 0.001

The addition of the pre-extraction step effectively eliminated sample burning, reducing sample loss to zero across all subsequent runs. Moreover, it resulted in a significant improvement in lipid recovery, especially for *Y. lipolytica*, from which the total FAME yields increased by an average of 70% compared to the original microwave protocol (Fig. 5A). A positive correlation was also observed for *B. adeninivorans*, as the mean of the total FAME content in this yeast increased on average by 32%, although only the increase in C18:0 content reached statistical significance (*p* < 0.05) (Fig. 5B). Notably, smaller standard deviations were obtained with the pre-extraction step in both yeast samples, resulting in decreased variability and suggesting that the bead-based pre-extraction step enhances both recovery and precision for the method. Importantly, the relative proportions of different FAMEs were consistent between the tested extraction methods, indicating that there was no compositional fatty acid bias introduced (Fig. 5C).

Since adding a bead-based pre-extraction step enhanced lipid recovery and precision, and avoided sample losses, it was concluded that the bead assisted pre-extraction step should be integrated into the microwave-assisted protocol.

### dIS methylation efficiency depends on the microwave method

**Fig. 6:**
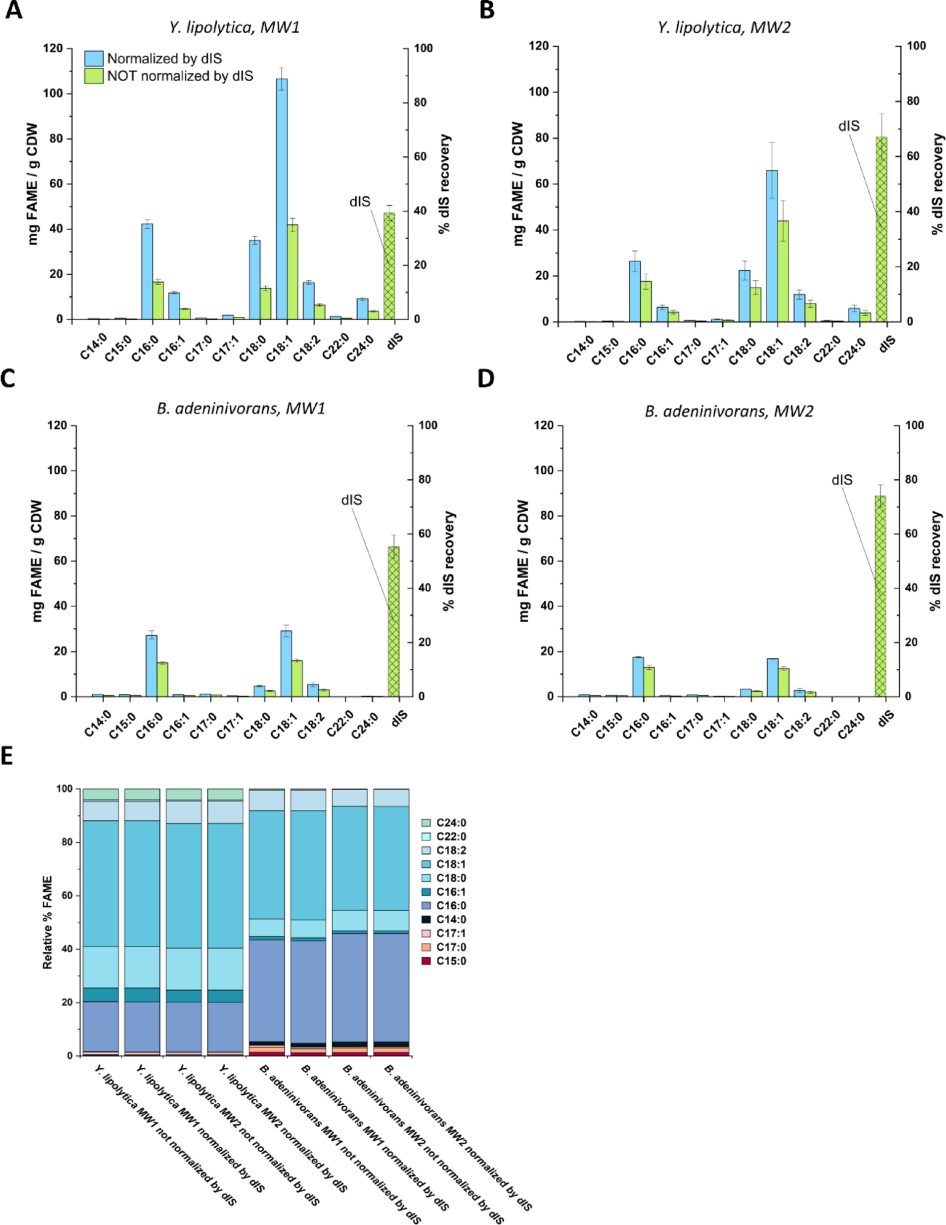
Comparison of transesterification efficiency at different microwave methods, with and without dIS normalization. Panels **A** and **B** show the amounts of ECFAs and OCFAs as FAMEs in *Y. lipolytica* before (green) and after (blue) dIS normalization. Panel A presents data from less-harsh microwave method (MW1), while Panel B presents data from the harsher microwave method (MW2). Panels **C** and **D** display the ECFA and OCFA levels as FAMEs in *B. adeninivorans*, also before (green) and after (blue) dIS normalization. Panel C corresponds to the less-harsh microwave method, and Panel D to the harsher microwave method. The y-axis represents the FAME yield in mg per g of CDW, and the x-axis shows the FAME profile. In panel **E** the relative percentage of FAMEs are shown for both strains and both microwave methods, before and after normalization by dIS

Accurate normalization and analysis by GC–MS depend on the dIS serving as a stable and reliable reference, by (i) remaining recoverable throughout the extraction process, (ii) undergoing methylation as efficient as the fatty acids in TAGs, and (iii) accounting for processing losses. However, if the methylation rate and efficiency of the dIS differs from that of the fatty acids in the sample, the final quantification and normalization may be affected. To assess the suitability of using non-methylated dISs for normalization when calculating FAME yields in the microwave-assisted protocol (MW1) (including the pre-extraction step), we first compared FAME yields calculating values both with and without normalization (see Eq. 2) to the added dIS. In both cases, yields were expressed relative to yeast biomass. Interestingly, we noticed that only 40% and 57% of the added dIS were recovered for *Y. lipolytica* and *B. adeninivorans*, respectively, which led to higher calculated FAME yields when normalization with dIS was applied compared to calculations without normalization (Fig. 6A, C).

To determine the fraction of dIS lost by physical loss during the procedure, we prepared samples containing only pre-methylated dIS and subjected them to the same treatment steps and microwave setting as the previous samples. On average, approximately 82% of the methylated dIS was recovered (data not shown), i.e. significantly more than the 40–57% recovery observed for the non-methylated dIS. This suggests that the low recovery of the non-methylated dIS is due to a combination of insufficient methylation and physical loss along the multi-steps protocol.

Furthermore, we investigated whether a more intensive microwave protocol (MW2) could enhance the efficiency of dIS methylation and/or TAG transesterification. Under the harsher conditions, 70–75% of the added dIS was recovered from both yeast strain samples, i.e. a significant increase compared to the original microwave conditions (40–57%), and approaching the recovery levels observed for the pre-methylated dIS (82%). However, the raw concentration of the FAMEs (in μg/mL) after calibration, adjusted only for the dilution factor and initial biomass, without normalization to the dIS found in the sample, showed no significant differences between the two microwave protocols (Fig. 6B, D). This suggests that the dIS undergoes esterification with different efficiencies in the two microwave protocols, whereas TAG conversion appears equally efficient in both. Importantly, the FAME profiles remained consistent throughout experiments (Fig. 6E), confirming the robustness and reproducibility of the qualitative profiling shown in Fig. 5.

Since the non-methylated dIS was not efficiently converted using the microwave-assisted protocol (with and without the pre-extraction step), it may have led to an overestimation of FAME yields reported in Fig. 5. Thus, we repeated the analysis with the microwave-assisted protocol including the pre-extraction step, this time using the pre-methylated dIS and a new batch of *Y. lipolytica* biomass. The improved extraction efficiency observed with the bead-based method was still evident, although to a lesser extent (Supplementary Fig. 1).

Overall, the results across the investigated protocols highlight that the choice of IS (non-methylated vs. pre-methylated) can differently impact normalization—unless an effective microwave protocol is found and applied to ensure complete methylation of the non-methylated dIS. Consequently, it´s of primary importance to highlight the necessity to carefully adapt the protocol when using a deuterated IS at any degree of substitution.

### The improved FAME protocol ensures reproducibility

Finally, we conducted an experiment to include all the improvements made so far and evaluate the precision of the optimized FAME protocol. Here, we incorporated the bead-based pre-extraction step, combined with the less-harsh microwave method and the pre-methylated dIS to avoid normalization uncertainties. At this point we also omitted the hexane evaporation and subsequent resuspension of the extracted FAMEs, as the methylated dIS was expected to compensate for any volume losses or solvent evaporation potentially occurring during microwave processing. Three independent FAME experiments and microwave runs were performed in two different days, each consisting of three technical replicates for each yeast strain. All samples were analyzed within the same GC/MS run.

The coefficient of variation (% CV) was calculated to determine the precision of the new established protocol, both among the technical triplicates within each run and all combined runs (Table 2). Additionally, the average % CV was calculated for the total FAME profile within each run and all combined runs. Except for run 2, which appeared to be an outlier in both strains, the overall % CV across batches was approximately 16% for *Y. lipolytica* and 4% for *B. adeninivorans,* respectively, indicating an acceptable degree of variability (Table 2) In addition, the technical triplicates within runs 1 and 3 for both strains exhibited low variability, with % CV values of approximately 8–10%. The high % CV observed in microwave run 2 for both strains (42.1% for *Y. lipolytica* and 19.9% for *B. adeninivorans*), were due to two outliers among the three technical replicates, which had lower yields as shown in Supplementary Fig. 2. While the FAME yields across the runs were not statistically significantly different, the mean value of run 2 was noticeably lower compared to other runs, indicating that the extraction and/or transesterification efficiency was reduced in some replicates during this run. The microwave-assisted method regulates applied wattage to maintain the programmed temperature and duration; however, we observed some variability in the final achieved temperature between runs (approximately ± 2 °C). Additionally, the position of individual vessels within the microwave chamber may influence heating, as temperature distribution is unlikely to be completely uniform. Together, these factors may contribute to reduced reproducibility in FAME yield, while not affecting the qualitative FAME profiles. This underscores the importance of including multiple technical replicates, performing different microwave runs and when appropriate, excluding clear outliers from the calculation of mean and standard deviation. The data also highlights how a single method can yield variable precision across two yeast strains. Nevertheless, the optimized protocol demonstrated high precision in both strains, making it a robust method for fatty acid analysis.

**Table 2:** % CV calculated among three different batches of experiments

		C14:0	C15:0	C16:0	C16:1	C17:0	C17:1	C18:0	C18:1	C18:2	C22:0	C24:0	Average
*Y. lipolytica*	1	6.2	10.6	5.2	4.0	5.7	21.3	7.6	5.9	4.4	7.4	12.7	8.3
	2	17.2	54.4	41.1	39.8	27.4	44.4	46.5	44.8	42.3	46.5	59.2	42.1
	3	19.4	24.5	4.2	4.2	8.8	4.6	4.3	3.7	4.6	2.2	6.2	7.9
	All runs	16.4	17.5	15.3	15.4	10.5	9.0	16.9	16.6	16.4	21.2	20.1	16.0
*B. adeninivorans*	1	7.8	12.6	10.3	8.3	13.8	8.5	13.3	11.6	10.7	N.A	N.A	10.8
	2	20.0	18.9	19.5	18.0	19.1	25.3	20.2	21.0	17.5	N.A	N.A	19.9
	3	7.5	2.7	6.9	6.5	7.9	11.2	7.1	9.2	10.1	N.A	N.A	7.7
	All runs	6.0	3.8	3.5	1.9	6.2	8.4	3.6	3.0	2.7	N.A	N.A	4.3

## Discussion

In this study, we tailored and optimized a FAME protocol for simultaneous analysis of ECFAs and OCFAs, employing a fully deuterated internal standard. By comparing the distantly related yeast species *Y. lipolytica* and *B. adeninivorans* as producers of both major and trace fatty acids, we observed species-specific differences in TAG extraction and protocol efficiency, likely due to differences in cell wall composition and TAG content. Our findings also suggest that the extraction and transesterification methods significantly influence the FAME yield and precision. Though this is not surprising as such, it highlights the necessity of thoroughly considering all aspects of the analytical procedure to ensure reliable results rather than using existing protocols optimized for general purpose FAME analysis.

Here, we evaluated two fundamentally different FAME protocols, referred to as the “shaker-assisted” and “microwave-assisted” protocols—both designed to perform extraction and transesterification simultaneously. Interestingly, the subsequent GC–MS analysis showed that the dIS was successfully recovered using the microwave-assisted protocol, whereas recovery was negligible with the shaker-assisted protocol. These results indicate that, under the current experimental conditions, the shaker-assisted protocol was an unsuitable option with the chosen dIS. The underlying reasons remain speculative. Under base-catalyzed conditions, TAGs are rapidly transesterified to FAME, whereas the free fatty acid internal standard is converted to its sodium salt, which only esterifies after acidification. If this step is brief, incomplete conversion may result. Other possibilities can be (i) destabilization of the dIS by the harsh basic (NaOH) and acidic (H₂SO₄) conditions, leading to its decomposition or the formation of non-volatile by-products, (ii) the methylating agents might have reacted inefficiently with the heavily deuterated molecule. Although deuterium loss through D–H exchange is another potential explanation, no additional peaks were observed in the total ion chromatograms, suggesting that such exchange did not occur. Further investigation would be required to clarify the causative mechanism(s), which is beyond the scope of the present study.

Aiming to improve lipid extraction efficiency from yeast samples in the microwave-assisted protocol, we also assessed separation of the extraction and transesterification steps. The original protocol was compared with a modified version, including a bead-based pre-extraction step, to physically disrupt cells through vigorous mixing, enhancing access to non-polar lipids by breaking down thick cell walls and lipid bodies where TAGs are stored. Our results showed that this step significantly improved extraction efficiency, increasing yields for all FAMEs in the profile. It also reduced variability among replicates, making the protocol more precise, opposite to the findings described by Khoomrung and colleagues (Khoomrung et al. 2013). However, the effectiveness of the extraction appears to be species-dependent, with differing impacts observed between the two yeast species tested. This may be explained by the fact that yeasts’ cell walls are complex and thick structures composed of oligosaccharides, mannoproteins, chitin and lipids, with composition varying greatly across species (Jacob 1992). In addition, the extracted sample matrices are often highly heterogeneous, containing diverse lipids, proteins, and oligosaccharides in varying proportions. Thus, no universal extraction method suits all species, and this should be considered when comparing fatty acid yields across different yeasts, as extraction efficiency, and thus accuracy, may vary between strains. However, the qualitative FAME profiles were highly reproducible and remained consistent regardless of the applied FAME protocol.

Moreover, in the microwave-assisted protocol that included a bead-based pre-extraction step, we observed that the fully deuterated free fatty acid IS (myristic acid-d₂₇) showed lower conversion efficiency to FAME compared with TAG-derived fatty acids. This likely reflects differences in reaction pathways. In acid-catalyzed protocols, the internal standard is introduced in hexane, which can limit contact with the methanolic catalyst and hinder esterification relative to lipids solubilized within the sample (Ichihara and Fukubayashi 2010). Transesterification of TAGs likely occurs efficiently at the phase interface, while esterification of free fatty acids depends on their solubility in methanol. Poor mixing or limited interface transfer can slow dIS conversion. To examine the effect of dIS conversion efficiency based on method parameters, we compared two microwave-assisted methods with different temperature ramps, one less intensive than the other. The less intense method was sufficient for complete TAG transesterification, as indicated by comparable FAME titers between methods prior to dIS normalization. In contrast, esterification of the dIS varies markedly between the methods. Thus, a complete esterification of the dIS requires either longer reaction times and/or elevated temperatures. Additionally, we observed that dIS recovery varied between yeast species, suggesting that differences in TAG content could influence the dIS reaction, as TAGs compete for the same reagents. Together, these factors suggest that deuterated free fatty acid standards may not fully mimic TAG behavior during derivatization and support the use of pre-methylated internal standards for improved yield recovery in this application.

## Conclusions

Our study led to a tailored and optimized protocol for FAME analysis of oleaginous yeast, including both ECFAs and OCFAs. The most important changes made to the original protocol relate to the internal standard used and the normalization employed, while the most important improvements relate to the extraction procedure and the increased resolution in the GC-analysis. Our findings underscore that FAME protocols can yield variable results across different yeast species, emphasizing the need to tailor extraction methods to the specific biological context—particularly considering differences in cell wall composition and intracellular lipid content. Additionally, although normalization with ISs is essential for accurate quantification, it can introduce uncertainty if the IS behavior is different from the analytes during extraction or transesterification. In this study, the fully deuterated, non-methylated IS (myristic acid-d₂₇) proved challenging to use under the examined conditions, highlighting the importance of carefully adapting the FAME protocol upon employing deuterated standards at any level of substitution. However, by using a pre-methylated dIS to avoid normalization issues, we could confirm that the developed bead-supplemented microwave protocol offers improved FAME yields and reproducibility across both yeast strains.

## Supplementary Information

Below is the link to the electronic supplementary material.

Supplementary Material 1

## Data Availability

The datasets generated and analyzed in the study are available from the corresponding author on reasonable request.

## Abbreviations

OCFA: Odd-chain fatty acid
ECFA: Even-chain fatty acid
TAGs: Triacylglycerols
GC–MS: Gas chromatography-mass spectrometry
MeOH: Methanol
dIS: Deuterated internal standard
FA: Fatty acid
FAME: Fatty acid methyl ester
TIC: Total ion chromatogram
ES: External standards
IS: Internal standard
MW: Microwave
CDW: Cell dry weight
TE: Transesterification
