# Supplementary material for: Optimization of a fatty acid methyl ester protocol for quantification of odd- and even-chain fatty acids in yeast
Source: AMB Express. 2026 Feb 9;16:23. doi: 10.1186/s13568-026-02022-8 (PMC12932751; doi:10.1186/s13568-026-02022-8)
Supplement: Supplementary file 1 — Supplementary Material 1 [file 13568_2026_2022_MOESM1_ESM.docx]

Supplementary Material

**
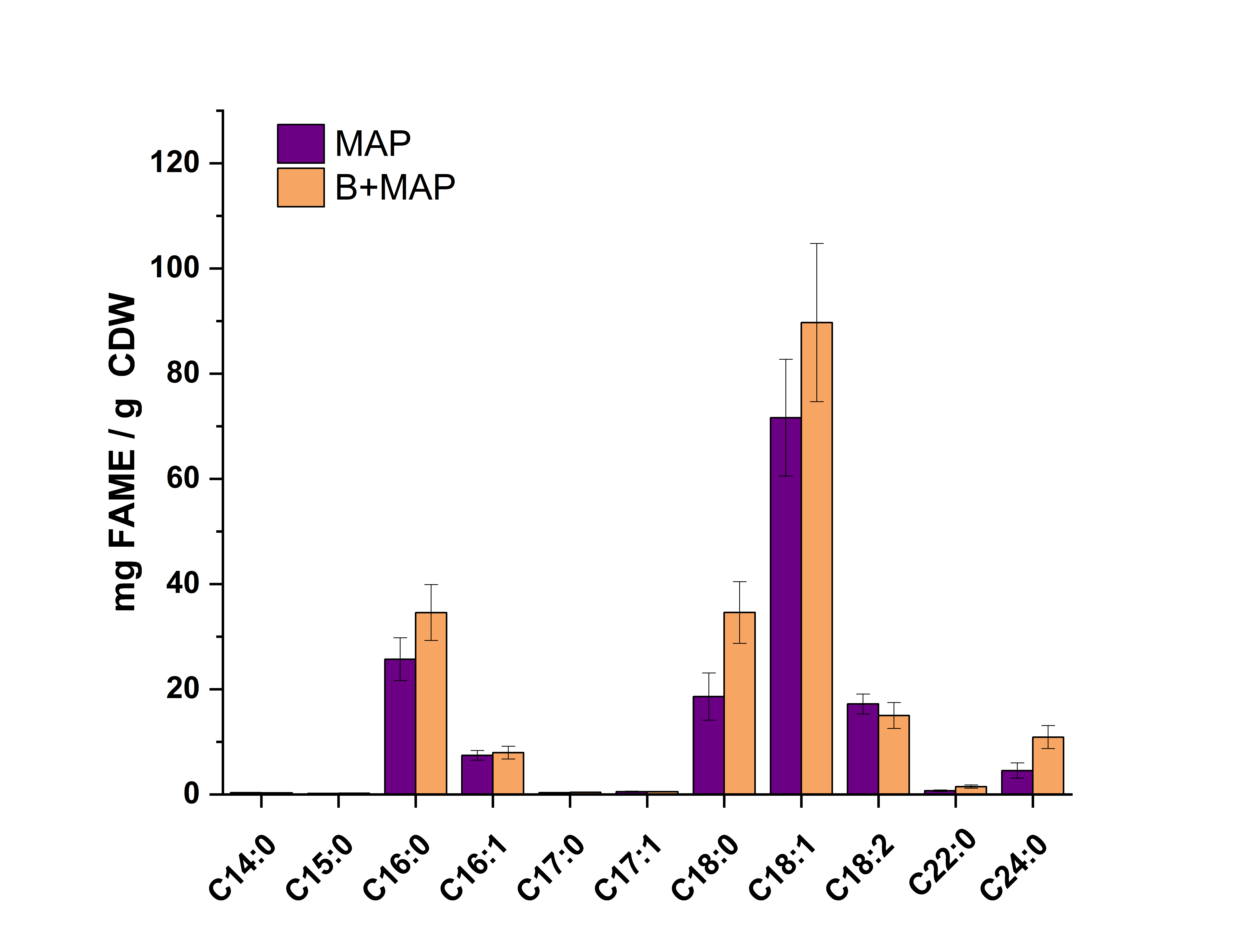
**

**Supplementary Figure 1. Comparison of extraction efficiencies with and without beads addition with pre-methylated dIS in *Y. lipolytica*.** The x-axis displays the FAME identified in the yeast profile, while the y-axis illustrates their concentrations in mg per g of CDW. The microwave-assisted protocol (MAP) is shown in purple, and the modified protocol incorporating bead-assisted pre-extraction (B+MAP) is shown in orange. The results show that the increase in extraction efficiency observed in Figure 5 decreased on average from 70% to 31%, likely due to an overestimation by dIS normalization caused by incomplete conversion of the non-methylated dIS used in that experiment.


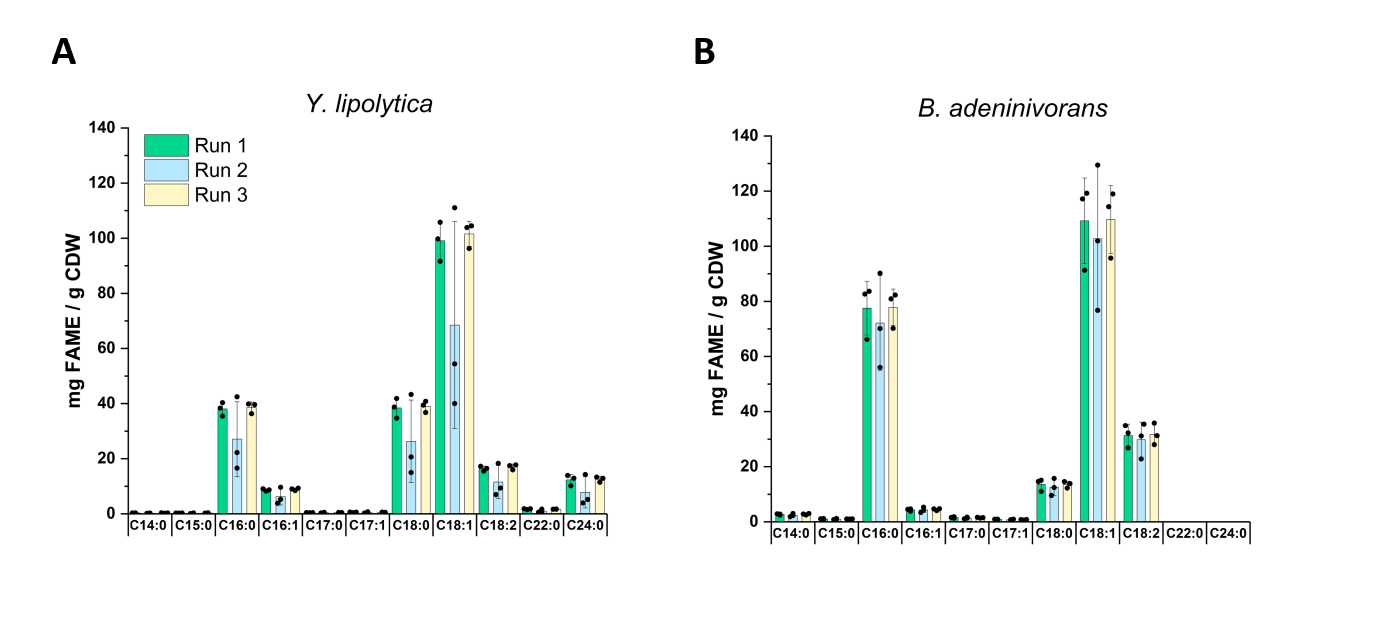


**Supplementary Figure 2. FAME yields from reproducibility test.**

Panel **A** displays *Y. lipolytica* and panel **B** *B. adeninivorans*. The x-axis in A and B displays the FAME identified in the yeast profiles, while the y-axis shows their yield in mg per g of CDW. In both panels, runs shown in the same color were processed together in the same microwave cycle, with each run consisting of three technical replicates. The T-test confirmed that there is no significant difference in fatty acid yields among the three runs for both strains.
